# New York State Veterinary Medical Society

**Published:** 1901-10

**Authors:** 


### SOCIETY PROCEEDINGS.

### NEW YORK STATE VETERINARY MEDICAL SOCIETY.

The eleventh annual meeting of the New York State Veterinary Medical Society took place September 10th and 11th at Ithaca, N. Y., in the lecture-room of the New York State Veterinary College.

On September 10th, at nine o'clock, a clinic was held; at eleven o'clock the meeting was convened by the President, R. R. Bell. In the absence of the President of Cornell University, the address of welcome was made by Prof. James Law. The President, Dr. Bell, responded in a few words and then gave his annual address.

Roll-call was omitted, as a register had been placed at the door for signatures of those in attendance.

The President suggested dispensing with the minutes; Dr. H. D. Hanson requested the reading of the minutes; after a whispered conversation by the Secretary, Dr. Claude D. Morris, with the President, the President announced that the Secretary had not brought the minutes of the Society with him. On motion, the minutes were dispensed with.

The report of the Secretary was read, calling attention to the delinquency in payment of dues and complaining of the small amount and slow collection of money for his own salary. He reported the resignation of Dr. John A. Bell from the State Board of Veterinary Examiners.

The Board of Censors reported favorably the names of seven applicants who were elected to membership.

The Executive Committee then reported the minutes of two meetings (See page 646, this JOURNAL).

On motion, the charges against Claude D. Morris were referred to the Society.

On motion, the second section of the Executive Committee's report was finally adopted, and consideration of the charges against Claude D. Morris was deferred until the next annual meeting.

Papers were read as follows:

"Rabies," by Prof. James Law, by title. "The Importance of Rectal Explorations and Manipulation," by Dr. J. W. Corrigan.

At 8 P.M. an illustrated lecture on "Specific Etiology and Some of Its Problems," by Prof. V. A. Moore, proved extremely interesting, after which a handsome entertainment by the Faculty of the New York State Veterinary College was given.

On September 11th, after a clinic of three hours, the meeting convened.

Case reports were made as follows:

"A Peculiar Disease of Cattle," W. H. Phyfe.

"Severing of the Rib, with Protrusion of Omentum," W. H. Salisbury.

"Supernumerary Digits in a Foal," J. L. Wilder.

The Exhibition of Pathological Specimens, with Remarks, V. A. Moore and S. H. Burnett.

Papers were read as follows:

"The Influence of Altitude on the Results of Surgical Operations," by J. A. McCrank.

"The Tuberculin-test as a City Health Ordinance," by C. H. Jewell.

"How to Detect Bob-veal," by W. H. Kelly.

"Some Azoturia Experiences," by C. J. Mulvey, by title.

"An Abnormal Oviduct in an Adult Chick," by G. S. Hopkins.

"Spaying as a Remedy for Vice in Mares," by A. H. Ide. (See this JOURNAL, page 640.)

"Anthrax," discussed by C. D. Morris and V. A. Moore.

"Development of the Metacarpus and Metatarus of Sheep," illustrated by gross and microscopical specimens, by Simon H. Gage.

"A Reliable Test for the Determination of Phosphates in the Urine of the Horse," by Pierre A. Fish.

"Treatment of Goitre in Dogs," by Pierre A. Fish.

"Fracture of the Tibia," by E. H. Nodine.

"Intestinal Obstruction," by S. R. Webber.

(These papers will appear later in the JOURNAL).

Brooklyn, N. Y., was selected as the place of the next annual meeting. The new President, Prof. James Law, was introduced by the retiring President, Dr. R. R. Bell.

A vote of thanks was extended to Prof. Law for the hospitality of the college.

Adjourned.

### OFFICERS.

Elected for coming year, 1901–1902.

*President.*—Prof. James Law, Ithaca, N. Y.

*Vice-President.*—James L. Robertson, 409 Ninth Avenue, New York City.

*Secretary-Treasurer.*—Wm. Henry Kelly, 195 Western Avenue, Albany, N. Y.

*Censors.*—Charles Cowie, Ogdensburg, N. Y., *Chairman ;*
E. B. Ackerman, Brooklyn, N. Y., H. D. Gill, New York, N. Y.,
Harry Sutterby, Batavia, N. Y., E. B. Ingalls, Mohawk, N. Y.

### State Examining Board.

The following were recommended to the Governor for selection of one to fill the vacancy caused by the resignation of John A. Bell, of Watertown: E. B. Ackerman, of Brooklyn; H. B. Stebbins, of West Winfield.

### Committees Appointed by the President.

(Not yet announced.)

### ATTENDANCE.

The following were present:

Drs. E. B. Ackerman, Brooklyn; Samuel Atchison, Brooklyn; W. C. Babcock, Scott; A. M. Baker, Brasher Falls; Roscoe R. Bell, Brooklyn; George H. Berns, Brooklyn; E. F. Bettinger, Chittenango; Wm. W. D. Bird, Buffalo; E. M. Casey, Oxford; C. E. Clayton, New York; J. W. Corrigan, Batavia; Charles Cowie, Ogdensburg; J. M. Currie, Rome; W. G. Dodds, Canandaigua; Alexander Findley, Camden; Pierre A. Fish, Ithaca; Simon H. Gage (Honorary), Ithaca; J. A. Genung, Ithaca; Harry D. Gill, New York; W. J. Hallock, Auburn; E. Hanshew, Brooklyn; H. D. Hanson, New York; G. S. Hopkins, Ithaca; Wilson Huff, Rome; R. S. Huidekoper, Philadelphia, Pa.; A. H. Ide, Lowville; E. B. Ingalls, Mohawk; Charles H. Jewell, Dunkirk; W. J. Johnson, Geneva; W. H. Kelly, Albany; G. C. Kesler, Holly; F. L. Kilborne, Kelloggsville; E. O. Kingman, Courtland; James Law, Ithaca; Veranus A. Moore (Honorary), Ithaca; R. W. McCully, New York; E. H. Nodyne, Sodus; T. F. O'Dea, Saugerties; Romanzo Perkins, Warsaw; W. H. Phyfe, Delhi; T. S. Rich, Avon; J. S. Robertson, New York; W. H. Salisbury, Clifton Springs; J. B. Sheaner, Pittsford; Thomas G. Sherwood, New York; H. D. Stebbins, West Winfield; Harry Sutterby, Batavia; W. B. Switzer, Oswego; A. J. Trexill, Auburn; E. F. Vorhis, Owego; L. R. Webber, Rochester; H. S. Wende, Buffalo; J. W. Werner, Lyons; A. G. Wicks, Schenectady; J. S. Wilder, Akron; W. L. Williams, Ithaca; Wm. F. Woolston, Fishers; J. H. Youngs, Belvidere.

The following visitors were present:

Professor Hughes, Chicago, Ill.; S. Stewart, the Secretary of the A. V. M. A., Kansas City, Mo.; S. D. Brimhall, Minneapolis, Minn.; Wm. Herbert Lowe, Paterson, N. J.; S. Brenton, Detroit, Mich.; Benj. D. Pierce, Springfield, Mass.; L. A. Merrillat, Chicago, Ill.

The following ladies attended the opening of the meeting and the social functions: Mrs. Bell, Brooklyn; Mrs. Berns, Brooklyn; Miss Berns,

Brooklyn; Mrs. Hanson, New York; Miss Huff, Rome; Mrs. Hughes, Chicago, Ill.; Mrs. Kelly, Albany; Mrs. Merillat, Chicago, Ill.; Mrs. Phyfe, Delhi; Mrs. Stewart, Kansas City, Mo.

### New Members Elected.

Charles E. Clayton, 207 W. Fifty-fourth Street, New York City; A. Barradel, Pawling; J. F. Devine, Rhinebeck; G. S. Hopkins, Ithaca; James L. Robertson, New York City; Robert W. McCully, New York City; Alfred James Trexill, Auburn; Joseph W. Turner, Lyons; W. J. Johnson, Geneva.

### RESOLUTIONS

Passed at the eighth annual meeting, at Ithaca, N. Y., September 11, 1901.

### President McKinley.

*Resolved*, That the New York State Veterinary Medical Society, assembled in annual meeting at Ithaca, N. Y., expresses its sense of horror at the murderous assault on William McKinley, the honored President of the United States, its warm sympathy with the sufferer, and its profound gratitude that the object of the assassin has failed to be accomplished, and that there is every reason to hope for a speedy and complete recovery of the chief ruler of the nation.

### State Veterinarian.

*Resolved*, That this Association urge upon the Commissioner of the Department of Agriculture of this State the advisability of the appointment of a State Veterinarian who would be the Chief of the Veterinary Bureau of the said Department of Agriculture; or, if this is impossible under the present laws, that this Association would request his aid in the passage of a law which would create such a bureau with a veterinary chief.

*Resolved further*, That the Secretary be empowered to communicate with the Commissioner of the Department of Agriculture, with this end in view.

### Army Veterinary Service.

Whereas, The United States army is the only army in the civilized world which has not an organized veterinary service giving rank to the veterinarian; and

Whereas, Notwithstanding the endeavors and labor of many years on the part of the veterinary profession throughout the country to obtain proper and suitable recognition and adequate graded rank in the United States army; and

Whereas, The American Veterinary Medical Association, in its recent meeting, resolved to continue its endeavors and labor until we meet with success; therefore, be it

*Resolved*, That we, the New York State Veterinary Medical Society, as a body and as individuals, promise our hearty co-operation and assistance to the Committee on Army Legislation of the American Veterinary Medical Association until we do obtain from Congress the establishment of an efficient veterinary service in the army and graded rank for the veterinarian, recognizing his position as an educated professional man.

### Charges Against the Secretary, Claude D. Morris.

Charges and specifications against Claude D. Morris, made to the New York Veterinary Medical Society by Thomas G. Sherwood:

Charge 1. That Claude D. Morris made use of the letter-head of the New York State Veterinary Medical Society and of his signature as Secretary in addressing a letter to the Secretary of War without the authority of the Society.

To wit: In that he wrote a letter, of which the following is a copy:

New York State Veterinary Medical Society,
Binghamton, N. Y., December 31, 1900.

Hon. Elihu Root,
Secretary of War, Washington, D. C.

My Dear Sir: The position you take in relation to the Veterinary Corps bill is quite in accord with the sentiments of many of the best veterinary practitioners in the country.

The profession at large is not at heart asking for the enactment of this measure. It is not clear to the minds of many the necessity for a veterinary bureau, while we all agree as recognizing the importance of a high-grade service for the cavalry. The profession all over the country has been urged by the gentlemen who are fathering the scheme to render every possible aid in the endeavor to obtain a favorable consideration by Congress, and no doubt much of the request in behalf of the bill from constituents of Senators and Representatives is in obedience to this solicitation.

Our Society, at its annual meeting, September 1st, did not consider this matter, although Dr. Huidekoper was present, and it is presumed he was there for the purpose of enlisting support, but learning there was some opposition, no doubt considered that no indorsement from the Society was safer than an average resolution.

I am not speaking for the Society as against the bill, but simply to say that the State Society is not on record in behalf of the bill. As a private practitioner I have written our Senator, Hon. Chauncey M. Depew, stating my objections to this measure.

With sincere consideration and esteem, I beg to be,
Very respectfully,
Claude D. Morris, V.S.,
Secretary.

Charge 2. That in the foregoing letter he misrepresented the facts, suppressed the truth, and gave a false impression to the views and wishes of the Society.

To wit: In that the Society previously, and to his knowledge, had once or more voted in favor of attaching veterinarians as commissioned officers to the army, and that a very large majority of the members of the Society were in favor of such measure.

Charge 3. Conduct prejudicial to the best interests of the Society and of the profession and to the efficiency of the army.

To wit: In this that he, the said Claude D. Morris, V.S., in writing the foregoing letter, opposed the bill then pending before Congress, and produced or tried to produce the impression that the bill was against the interests of the veterinary profession and of the army, and this without the knowledge or consent of this Society.

Charge 4. Conduct unbecoming a gentleman, a professional man, and a member of our Society.

To wit: In this that by writing the foregoing letter to the Secretary of War he endeavored to produce on the mind of the Secretary the impression that the bill referred to was not favored by the Society or by the members thereof; whereas, in point of fact, as stated, a large majority of the members desired the bill to pass, as he very well knew.

Respectfully submitted,

Thomas G. Sherwood.

